# Baseline resistance-guided therapy does not enhance the response to interferon-free treatment of HCV infection in real life

**DOI:** 10.1038/s41598-018-33367-1

**Published:** 2018-10-08

**Authors:** Luis M. Real, Juan Macías, Ana B. Pérez, Dolores Merino, Rafael Granados, Luis Morano, Marcial Delgado, María J. Ríos, Carlos Galera, Miguel G. Deltoro, Nicolás Merchante, Federico García, Juan A. Pineda

**Affiliations:** 1 Unidad Clínica de Enfermedades Infecciosas y Microbiología, Hospital Universitario de Valme, Sevilla, Spain; 2 Servicio de Microbiología, Complejo Hospitalario Universitario de Granada-Hospital PTS, Granada, Spain; 3 Instituto de Investigación Biosanitaria (IBS), Granada, Spain; 4 Unidad de Enfermedades Infecciosas, Hospital Juan Ramón Jiménez, Huelva, Spain; 5 Unidad de Enfermedades Infecciosas, Hospital Universitario de Gran Canaria Dr. Negrín, Las Palmas, de Gran Canaria Spain; 6 Unidad de Patología Infecciosa, Hospital Universitario Alvaro Conqueiro. Instituto de Investigación Galicia Sur, Vigo, Spain; 7 Servicio de Enfermedades Infecciosas, Hospital Regional de Málaga, Málaga, Spain; 8 Unidad de Enfermedades Infecciosas, Hospital Universitario Virgen Macarena, Sevilla, Spain; 9 Servicio de Medicina Interna, Unidad de VIH. Hospital General Universitario Virgen Arrixaca, Murcia, Spain; 10 Unidad de Enfermedades Infecciosas, Consorcio Hospital General Universitario de Valencia, Valencia, Spain

## Abstract

Hepatitis C virus (HCV) response to direct-acting antivirals (DAAs) may be influenced by the presence of resistance-associated substitutions (RASs). This study aimed to assess if NS5A baseline RAS-guided treatment enhances the rate of sustained viral response (SVR) in naïve HCV-infected patients in clinical practice. All HCV-infected patients who initiated treatment with interferon (IFN)-free DAA-based regimens between March 2016 and May 2017 in 17 Spanish hospitals and who had evaluable SVR 12 weeks (SVR12) after the end of therapy were included. Patients had to be DAA naïve, with the exception of sofosbuvir with/without IFN. In one hospital, participants received therapy guided by the presence of NS5A-RASs (RGT population). Patients enrolled in the remaining hospitals, without baseline RASs testing, constituted the control population. A total of 120 and 512 patients were included in the RGT and control populations, respectively. Nine (7.5%) individuals in the RGT population showed baseline NS5A-RASs. All of them achieved SVR12. The SVR12 rate in the RGT population was 97.2% (three relapses) whereas it was 98.8% (six relapses) in the control population (p = 0.382). Our findings suggest that testing for baseline NS5A-RASs in naïve HCV-infected patients does not enhance the rate of SVR to DAA-based IFN-free therapy in clinical practice.

## Introduction

Treatment of hepatitis C virus (HCV) infection using direct-acting antiviral (DAA) combinations achieves high cure rates. However, a proportion of patients ranging from 1% to 15%^1–3^ fail to achieve sustained virological response. Factors related to DAA treatment failure are HCV genotype, DAA regimen, liver disease severity and previous treatment experience. Accordingly, these factors are considered to select treatment schemes^4–6^.

According to data generated in phase 2 and 3 clinical trials, resistance of HCV to DAAs due to the presence of resistance-associated substitutions (RASs) in the viral genome, mainly those within the NS5A gene, also can influence treatment response^7,8^. For this reason, the AASLD-IDSA guidelines recommend testing for baseline NS5A RASs in specific DAA-naïve HCV-infected populations who are going to be treated with DAA-combinations containing a NS5A inhibitor^4^. In contrast, EASL guidelines do not recommend systematic baseline RASs testing in DAA-naïve patients; however, as AASLD-IDSA guidelines, if baseline RASs in NS5A are known, EASL also provides guided decision^5^.

In-house methods based in population sequencing techniques are commonly used for RASs testing. This circumstance could affect the reliability of results. In addition, these techniques are time consuming and they are not available in every hospital worldwide. All these facts are barriers that could delay the beginning of resistance-guided treatments in daily clinical practice.

There is limited information on the potential impact of baseline RASs testing on the outcome of HCV treatment in DAA-naïve patients in real life. Cento *et al*.^9^ reported 100% sustained virological response (SVR) rate in DAA-naïve patients undergoing treatment guided by baseline RASs in clinical practice. Nonetheless, they did not compare with a control population without RAS-guided treatment. To our knowledge, no study has specifically been designed to analyse if routine RASs testing may enhance the SVR rate achieved in naïve patients treated with IFN-free regimens in a real-life setting. In our study, we aimed to assess whether baseline RASs guided treatment increases the rate of SVR in naïve HCV-infected patients in daily clinical practice.

## Results

### Characteristics of the study population

A total of 129 HCV-infected patients fulfilled the criteria to be included as the RGT population. Among them, 5 (3.8%) voluntarily dropped out, 3 (2.3%) were lost to follow-up and 1 (0.8%) discontinued treatment due to adverse events. Therefore, 120 (93.0%) individuals constituted the final RGT population.

Regarding the control group, among a total of 525 HCV-infected patients who met the inclusion criteria, 11 (2.1%) voluntarily dropped out and treatment was discontinued in 2 (0.4%) patients due to adverse events. Accordingly, 512 (97.5%) individuals comprised the final control group.

The main characteristics of RGT and control populations are depicted in Table 1.

**Table 1:** Main characteristics of patients.

Variables	RGT* population n = 120	Control group n = 512	p-value
Age, years**	50 (47–54)	51 (46–54)	0.686
Male gender, n (%)	101 (84.2)	356 (69.5)	0.001
HIV coinfection, n (%)	75 (62.5)	251 (49.0)	0.008
Cirrhotics, n (%)	25 (20.8)	130 (25.4)^†^	0.291
Viral load >800000 IU/µL, n (%)	85 (70.8)	361 (71.2)^‡^	0.936
Pre-treated, n (%)	28 (23.3)	133 (26.0)	0.550
HCV genotype			0.249
1a, n (%)	50 (41.7)	171 (33.4)	
1b, n (%)	30 (25.0)	143 (27.9)	
1a/b, n (%)	0	19 (3.7)	
2, n (%)	0	7 (1.4)	
3, n (%)	17 (14.2)	75 (14.6)	
4, n (%)	23 (19.2)	94 (18.4)	
5, n (%)	0	2 (0.4)	
Indeterminate, n (%)	0	1 (0.2)	

^*^RGT: Resistance-guided treatment.

^**^Mean (Quartil 1 – Quartil 3).

^†^Among 511 individuals.

^‡^Among 507 individuals.

### Baseline RASs and resistance-guided treatment

Nine (7.5%) patients showed RASs in NS5A, including 2 (4.0%) of 50 harbouring GT1a, 4 (13.3%) of 30 infected with GT1b and 3 (17.6%) of 17 bearing GT3. No RASs were detected in GT4-infected patients and no patient showed more than one RASs (Table 2). Table 2 shows the DAA combinations used in each of these patients. Among the 9 patients with RASs, 8 (90.0%) received a resistance adapted DAA regimen. The remaining patient was GT1a-infected (Table 2, patient 1) with Y93L in NS5A, who was treated with sofosbuvir plus ledipasvir without ribavirin during 12 weeks due to drug-drug interactions issues. Specifically, this patient was being treated for HIV-infection with rilpivirine and it was not possible to change this treatment. Rilpivirin shows interactions with paritaprevir-ritonavir/ombitasvir, and therefore, these drugs could not be used. In addition, he was also intolerant to ribavirin and, consequently, it could not be administrated.

**Table 2:** Patients from the resistance-guided treatment who showed RASs in NS5A and DAA-based regimens used.

Patient	HCV genotype	NS5A RAS	Cirrhosis	DAA^*^ regimen	RBV^**^ use	Duration (Weeks)
1	1a	Y93L	No	SOF plus LDV	No	12
2	1a	M28V	No	PrOD	Yes	12
3	1b	P58S	No	PrOD	No	12
4	1b	L31V	No	PrOD	No	12
5	1b	L31M	No	PrOD	No	12
6	1b	L31M	Yes	PrOD	Yes	12
7	3	Y93H	Yes	SOF plus VEL	Yes	12
8	3	Y93H	Yes	SOF plus VEL	Yes	12
9	3	A30K	Yes	SOF plus DCV	Yes	24

^*^SOF: Sofosbubir; LDV: ledipasvir; DCV: daclastavir; PrOD: paritaprevir-ritonavir/ombitasvir plus dasabuvir; VEL: velpatasvir.

^**^RBV: ribavirin.

**Figure 1:**
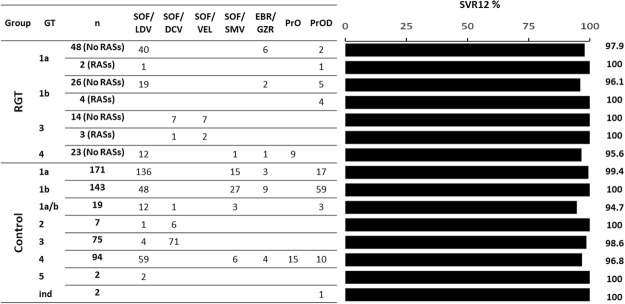
Treatments received and SVR12 rates. Number of treatments received and SVR12 rate achieved in accordance with HCV genotype and presence/absence of NS5A RASs in both the resistance-guided treatment population and control group. GT: Genotype; RGT: Resistance-guided treatment; SOF/LDV: sofosbubir/ledipasvir; SOD/DCV: Sofosbubir/daclastavir; SOF/VEL: Sofosbuvir/Velpatasvir; SOF/SMV: sofosbuvir/simeprevir; EBR/GZR: Elbasvir/grazoprevir; PrOD: paritaprevir-ritonavir/ombitasvir plus dasabuvir; PrO: paritaprevir-ritonavir/ombitasvir.

Table 3 and Fig. 1 shows the DAA-combinations used in both the RGT and the control populations. Globally, 30 (25.0%) patients in the RGT population and 99 (19.3%) in the control population received ribavirin (p = 0.166). Treatment duration was longer than 12 weeks for 3 (2.5%) and 20 (3.9%) patients in the RGT and control populations, respectively (p = 0.595). Regarding those GT1-infected individuals, 29 (36.3%) in the RGT population and 56 (11.4%) in the control population (p < 0.001) were treated during 8 weeks. These 8 weeks regimens were carried out among non-cirrhotic patients and were mainly based in sofosbuvir/ledipasvir (89.6%) or paritaprevir-ritonavir/ombitasvir plus dasabuvir (6.0%) combinations.

**Table 3:** DAA combinations used in the resistance-guide treatment population and in the control group.

Regimen^*^	RGT^**^ population n = 120	Control group n = 512
SOF plus DCV with/without RBV, n (%)	8 (6.7)	78 (15.2)
SOF plus SMV with/without RBV, n (%)	1 (0.8)	51 (10.0)
PrOD with/without RBV, n (%)	12 (10.0)	90 (17.6)
PrO with/without RBV, n (%)	9 (7.5)	15 (2.9)
SOF plus LDV with/without RBV, n (%)	72 (60.0)	262 (51.2)
SOF plus VEL with/without RBV, n (%)	9 (7.5)	0
EBR plus GZR, n (%)	9 (7.5)	16 (3.1)

^*^SOF: Sofosbubir; RBV: ribavirin; DCV: daclastavir; SMV simeprevir; PrOD: paritaprevir-ritonavir/ombitasvir plus dasabuvir; PrO: paritaprevir-ritonavir/ombitasvir; LDV: ledipasvir; VEL: Velpatasvir; EBR: Elbasvir; GZR: grazoprevir.

^**^Resistance-guided treatment.

### Impact of baseline RASs on SVR

Three out of 120 individuals relapsed in the RGT population (Fig. 1). None of them had baseline RASs and none of them was cirrhotic. They were infected with GT1a, GT1b, and GT4 (Supplementary Table S1). The remaining 117 (97.2%) subjects included in this population reached SVR12.

In the control group, six relapses were observed (one patient infected by GT1a, one with GT1 –unspecified subtype-, one bearing GT3 and three with GT4) (Fig. 1). Five of them were cirrhotic (Supplementary Table S1). The remaining 506 (98.8%) patients reached SVR12, rate that was not statistically different to that found in the RGT population (98.8% versus 97.5%, p = 0.382).

Supplementary Table S2 depicts details about the SVR12 rates according to the specific regimens used and cirrhosis status in patients infected by GT1a, GT1b, GT3 and GT4 in both RGT and control groups. In addition, Supplementary Table S3 shows the global SVR12 rates and the main characteristics of patients according to those HCV genotypes.

### Emergent RASs after DAA-treatment in RGT population

Two (66.6%) of three patients who failed to the HCV treatment in the RGT population showed RASs in NS5A after treatment. Specifically, a GT4-infected patient that had been treated with sofosbuvir/ledipasvir during 12 weeks and a GT1b-infected individual who was treated with ombitasvir/paritaprevir/dasabuvir for 12 weeks showed the substitutions Y93L and M28T, respectively.

## Discussion

Our results suggest that routine testing of baseline RASs in naïve HCV-infected patients does not enhance the rate of SVR to all oral DAA-based therapy in daily clinical practice. These findings support EASL recommendations^5^. In fact, the rate of SVR achieved by non-guided treatment is so high, that no benefit is added by RAS testing.

Data derived from phase 2 and 3 clinical trials showed that some RASs in NS5A impact the SVR rate of patients under particular combinations, mainly in those infected with GT1a or GT3, especially if they have cirrhosis and/or were previously treated with interferon based regimens^3,10–14^. According to AASLD guidelines^4^, this justifies baseline RAS testing in HCV patients who are going to be treated with specific combinations. However, taking into account that the proportion of individuals showing baseline NS5A RASs is estimated to be 15%^13^, and that the reduction in SVR rates they cause is usually lesser than 10%, the determination of baseline RASs would avoid viral failure in less than 1% of naïve patients. In our population, the frequency of baseline NS5A RASs was 7.5%, which partly explains that determination of RASs did not lead to an increase in SVR rates. The low frequency of baseline NS5A RASs in Spanish patients, specifically those infected with GT1a, also has been observed by others^15,16^. Accordingly, Palladino *et al*.^15^ also suggested that in our country NS5A RASs testing is not necessary for HCV GT1a infected patients who are going to be treated with elbasvir^15^, a setting where the impact on SVR seems to be stronger^17^. In contrast, emerging RASs after the DAA treatment are detected in most non-SVR patients^18^. In our study population, two of the three relapses showed RASs in NS5A at failure. This fact supports RAS testing in DAA-experienced patients before re-treating as it has been proposed^18^.

Recently, a real-life study reported how 130 DAA naïve HCV-infected patients who received baseline RASs guided therapy achieved 100% of SVR12^9^. The authors concluded that this strategy could help to minimize or eliminate relapses. However, in this study, no control group of patients in whom baseline RASs were not investigated was included. Therefore, and in contrast to our work, it was not possible to test whether or not the treatment guided by baseline RASs really impacts the SVR rate. In addition, and taking into account that in our RGT population relapses were only observed in patients without baseline RASs, we point out that the baseline RASs testing does not completely prevent relapses. Supporting this, in a retrospective Spanish study carried out in real-life among patients who did not received a RASs-guided treatment^19^, only one of the RASs detected at baseline also appeared at relapse, whereas the rest of them were replaced with others at failure. Interestingly, more than half of the failures observed in naïve patients occurred in absence of baseline RASs^19^. These facts suggest a negligible effect of these variants in the outcome of the treatment of naïve patients at clinical practice. Therefore, the genetic barrier of the DAA combinations would be high enough to overcome the resistance caused by these RASs. This is probably another reason why we did not find differences in the SVR rate obtained in the group of patients with treatment guided by RASs and in the group of patients in whom RASs were not conducted.

In this study, we did not include the analysis of RASs in the NS3 gene. The clinical relevance of these RASs in naïve patients is lower than that found for NS5A. Moreover, almost all of them also show a lower frequency at baseline^8,20^. The exception is the Q80K which frequency in Europe is about 19%^20^. This variation has been associated with high resistance to simeprevir in GT1a infected patients receiving simeprevir along with pegylated IFN and RBV^21^. However, current evidence does not support a substantial effect of this variant on responses to treatment with simeprevir plus sofosbuvir in naïve patients without cirrhosis^14^, although it could reduce the rate of SVR in cirrhotics^22^. Nevertheless, this DAA combination was uncommonly used in our population.

This study has several limitations. First, the comparative analysis of SVR rates was carried out on groups of patients that were not randomized. Therefore, the existence of bias cannot be discarded. However, although some differences were found in the proportion of male gender and HIV-coinfection between the RGT and the control populations, none of them has a major impact on the likelihood of SVR to IFN-free DAA combinations. Similarly, there were significant differences in the proportion of non-cirrhotic GT1-infected patients treated during 8 weeks in the RGT and control populations. Nonetheless, non-inferiority of sofosbuvir/ledipasvir or paritaprevir-ritonavir/ombitasvir plus dasabuvir for 8 weeks compared to 12 weeks regimens has been reported^23–25^. Therefore, and due to the high rates of SVR observed in both populations, it is unlikely that a randomized study would find significant differences in SVR rates between those groups of patients. Secondly, in our study there were a low number of patients treated with specific combinations for which the presence of RASs leads to a reduction of SVR rates higher than 20% in phase 2 and 3 studies. This is the case of those GT1a infected individuals treated with elbasvir/grazoprevir, where the presence of specific baseline RASs to elbasvir reduced the SVR rate to 58%^17^. Therefore, we cannot discard the utility of the determination of RASs in specific groups of naïve individuals, such as subjects with GT1a who are going to receive elbasvir/grazoprevir and probably also cirrhotic patients with GT3 receiving sofosbuvir/velpatasvir^3^. In spite of this, our study does not support a benefit of the routine determination of RASs in all naïve patients, at least in our geographical area. In addition, the new pangenotypic and high genetic barrier DAA combinations such as glecaprevir/pibrentasvir^26^ and sofosbuvir/velpatasvir/voxilaprevir^27^, which have proved to be extremely efficacious even in subjects with NS5A RASs, will probably make RASs detection unnecessary at any scenario.

In summary, we compared the SVR rate achieved in a group of patients with treatment-guided by baseline RAS testing with that found in a control group of patients in whom baseline RASs were not determined. Although all individuals carrying baseline RASs reached SVR in the group of patients with guided treatment, the SVR rate in this group was not statistically different to that observed in the control group. Moreover, the few relapses observed in the group of patients with guided-treatment by RASs were not driven by the presence of RASs. These results suggest that baseline RASs determination in naïve patients does not prevent HCV treatment failure in the clinical practice.

## Methods

### Patients and study design

This is a prospective study conducted in two cohorts of DAA-treated patients attending Infectious Diseases Units of 17 hospitals across Spain: the HEPAVIR (clinicaltrials.gov ID: NCT02057003) cohort, which recruits HIV/HCV-coinfected patients and the GEHEP-MONO (clinicaltrials.gov ID: NCT02333292), which enrols HCV-monoinfected patients^28^. As inclusion criteria in this study, all individuals participating in these cohorts who initiated treatment with all-oral DAA-based regimens between March 2016 and May 2017, and who had never been treated before with DAAs, with the exception of sofosbuvir with or without ribavirin alone or plus interferon, were included.

RASs testing was available for patients from one of the participant hospitals. In these particular individuals, HCV treatment was selected taking into account the presence of baseline RASs in NS5A –resistance-guided treatment (RGT) population-. The individuals enrolled in the remaining hospitals, where baseline RASs determination was not conducted, were considered as control population.

### Treatment regimens and follow up

DAA regimen was decided according to the DAA available in the study period, the potential for interactions with concomitant medications, the severity of liver disease and comorbidities, following Spanish guidelines^6^. The treating physician took the final decision on individual DAA regimens. In the RGT population, the specific anti-HCV DAA combination was also decided on the basis of the presence of baseline RASs, whenever other potential issues (e.g. drug-drug interactions) allowed it. Thus, patients with RASs in NS5A were treated with a DAA combination containing a protease inhibitor when possible, or, alternatively, adding ribavirin and/or extending treatment.

Plasma viral load was evaluated at baseline and, at least, at the end of therapy and at week 12 post-treatment. SVR12 was defined as undetectable plasma HCV RNA 12 weeks after the end of therapy.

### RASs testing

Total RNA was isolated from plasma samples using the MagNA Pure Compact (Roche Diagnostics, Basel, Switzerland) and cDNA was synthesized by means of RevertAid H Minus First Strand cDNA Synthesis Kit (Thermo Fisher Scientific, Waltham, Massachusetts, EEUU). The NS5A gene was sequenced by standard Sanger techniques, using *in house* developed assays covering codons 1–99^29^. A sensitivity of approximately 15–20% can be assumed for this method^30^. RASs were considered clinically relevant according to that proposed elsewhere^7,31^.

### Statistical analysis

The primary outcome variable was SVR12 evaluated on an on-treatment (OT) approach, i.e, excluding patients with premature discontinuations or with missing SVR data. Comparisons were performed applying the Mann-Whitney-U test for continuous variables and the Chi-square or Fishers test, when necessary, for categorical variables.

Data were analysed using IBM SPSS 23.0 version (IBM Corporation, Somers, NY, USA).

### Ethics

This study was designed and performed according to the Helsinki declaration and was approved by the “Comité de Etica de la Investigación Sevilla Sur del Hospital Universitario de Valme” (Seville, Spain) and by the “Comité de Etica de la Investigación de la Provincia de Granada, Hospital Universitario San Cecilio” (Granada, Spain). All patients gave their written informed consent before entering the cohorts.

## Electronic supplementary material

Supplementary Tables

## Data Availability

All data generated or analysed during this study are included in this published article (and its Supplementary Information files).

## References

[1] Pawlotsky JM (2014). New hepatitis C therapies: the toolbox, strategies, and challenges. Gastroenterology.

[2] Pawlotsky JM (2015). Hepatitis C treatment: the data flood goes on-an update from the liver meeting 2014. Gastroenterology.

[3] Curry MP (2015). Sofosbuvir and Velpatasvir for HCV in Patients with Decompensated Cirrhosis. N Engl J Med.

[4] AASLD-IDSA. Recommendations for Testing, Managing, and Treating Hepatitis C. http://www.hcvguidelines.org.

[5] EASL. Recommendations on Treatment of Hepatitis C 2016, Update of September 2016. http://www.easl.eu/medias/cpg/HCV2016/English-report.pdf (2016).

[6] AEEH/SEIMC. Guías de manejo de la Hepatitis C. http://aeeh.es/wp-content/uploads/2017/06/consenso.pdf (2017).

[7] Pawlotsky JM (2016). Hepatitis C Virus Resistance to Direct-Acting Antiviral Drugs in Interferon-Free Regimens. Gastroenterology.

[8] Sarrazin C (2016). The importance of resistance to direct antiviral drugs in HCV infection in clinical practice. J Hepatol.

[9] Cento V (2017). Optimal cure rate by personalized HCV regimens in real-life: a proof-of-concept study. J Antimicrob Chemother.

[10] Foster GR (2015). Sofosbuvir and Velpatasvir for HCV Genotype 2 and 3 Infection. N Engl J Med.

[11] Jacobson IM (2017). Safety and Efficacy of Elbasvir/Grazoprevir in Patients With Hepatitis C Virus Infection and Compensated Cirrhosis: An Integrated Analysis. Gastroenterology.

[12] Nelson DR (2015). All-oral 12-week treatment with daclatasvir plus sofosbuvir in patients with hepatitis C virus genotype 3 infection: ALLY-3 phase III study. Hepatology.

[13] Zeuzem S (2017). NS5A resistance-associated substitutions in patients with genotype 1 hepatitis C virus: Prevalence and effect on treatment outcome. J Hepatol.

[14] Kwo P (2016). Simeprevir plus sofosbuvir (12 and 8 weeks) in hepatitis C virus genotype 1-infected patients without cirrhosis: OPTIMIST-1, a phase 3, randomized study. Hepatology.

[15] Palladino C (2017). Low frequency of NS5A relevant resistance-associated substitutions to Elbasvir among hepatitis C virus genotype 1a in Spain: a cross-sectional study. Sci Rep.

[16] Pérez, A. B. *et al*. In III Congreso Nacional del Grupo de Estudio de las Hepatitis Víricas (GEHEP) de la SEIMC 4 (Enferm Infecc y Microbiol Clín, Sevilla, 2017).

[17] Zeuzem S (2015). Grazoprevir-Elbasvir Combination Therapy for Treatment-Naive Cirrhotic and Noncirrhotic Patients With Chronic Hepatitis C Virus Genotype 1, 4, or 6 Infection: A Randomized Trial. Ann Intern Med.

[18] Halfon Philippe, Scholtès Caroline, Izopet Jacques, Larrat Sylvie, Trimoulet Pascale, Zoulim Fabien, Alric Laurent, Métivier Sophie, Leroy Vincent, Ouzan Denis, de Lédinghen Victor, Mohamed Sofiane, Pénaranda Guillaume, Khiri Hacène, Thélu Marie-Ange, Plauzolles Anne, Chiche Laurent, Bourlière Marc, Abravanel Florence (2017). Baseline and post-treatment hepatitis C NS5A resistance in relapsed patients from a multicentric real-life cohort. Antiviral therapy.

[19] Carrasco I (2017). Baseline NS5A Resistance Associated Substitutions May Impair DAA Response in Real-World Hepatitis C Patients. J Med Virol.

[20] Lenz, O. *et al*. Virology analyses of HCV isolates from genotype 1-infected patients treated with simeprevir plus peginterferon/ribavirin in Phase IIb/III studies. *J Hepatol* **62**, 10.1016/j.jhep.2014.11.0321008-1014 (2015). 10.1016/j.jhep.2014.11.032 25445400

[21] Verbinnen T (2015). *In Vitro* Activity of Simeprevir against Hepatitis C Virus Genotype 1 Clinical Isolates and Its Correlation with NS3 Sequence and Site-Directed Mutants. Antimicrob Agents Chemother.

[22] Lawitz E (2016). Simeprevir plus sofosbuvir in patients with chronic hepatitis C virus genotype 1 infection and cirrhosis: A phase 3 study (OPTIMIST-2). Hepatology.

[23] Buggisch P (2018). Real-world effectiveness of 8-week treatment with ledipasvir/sofosbuvir in chronic hepatitis C. J Hepatol.

[24] Kowdley KV (2014). Ledipasvir and sofosbuvir for 8 or 12 weeks for chronic HCV without cirrhosis. N Engl J Med.

[25] Welzel TM (2017). Ombitasvir, paritaprevir, and ritonavir plus dasabuvir for 8 weeks in previously untreated patients with hepatitis C virus genotype 1b infection without cirrhosis (GARNET): a single-arm, open-label, phase 3b trial. Lancet Gastroenterol Hepatol.

[26] Forns X (2017). Glecaprevir plus pibrentasvir for chronic hepatitis C virus genotype 1, 2, 4, 5, or 6 infection in adults with compensated cirrhosis (EXPEDITION-1): a single-arm, open-label, multicentre phase 3 trial. Lancet Infect Dis.

[27] Bourliere M (2017). Sofosbuvir, Velpatasvir, and Voxilaprevir for Previously Treated HCV Infection. N Engl J Med.

[28] Neukam K (2017). Liver stiffness predicts the response to direct-acting antiviral-based therapy against chronic hepatitis C in cirrhotic patients. Eur J Clin Microbiol Infect Dis.

[29] Barlett SR (2017). Sequencing of hepatitis C virus for detection of resistance to direct-acting antiviral therapy: A systematic review. Hepatology Communications.

[30] Dietz J (2015). Consideration of Viral Resistance for Optimization of Direct Antiviral Therapy of Hepatitis C Virus Genotype 1-Infected Patients. PLoS One.

[31] Lontok E (2015). Hepatitis C virus drug resistance-associated substitutions: State of the art summary. Hepatology.

