# Supplementary material for: Baseline resistance-guided therapy does not enhance the response to interferon-free treatment of HCV infection in real life
Source: Sci Rep. 2018 Oct 8;8:14905. doi: 10.1038/s41598-018-33367-1 (PMC6175866; doi:10.1038/s41598-018-33367-1)
Supplement: Supplementary file 1 — Supplementary Tables [file 41598_2018_33367_MOESM1_ESM.pdf]

**Baseline resistance-guided therapy does not enhance the response to interferon-free treatment of HCV infection in real life**”

Authors: Luis M Real, Juan Macías, Ana B Pérez, Dolores Merino, Rafael Granados, Luis Morano, Marcial Delgado, María José Ríos, Carlos Galera, Miguel G Deltoro, Nicolás Merchante, Federico García, Juan A Pineda

**Supplementary table 1.** Patients who relapsed.

| Patient | Group | HCV genotype | Age (years) | Cirrhosis | DAA regimen | RBV use | Duration (weeks) |
|---|---|---|---|---|---|---|---|
| **1** | **Control** | 1a | 52 | Yes | SOF/LDV | Yes | 12 |
| **2** | **Control** | 1a/b | 44 | Yes | SOF/DCV | Yes | 12 |
| **3** | **Control** | 3 | 52 | Yes | SOF/DCV | Yes | 12 |
| **4** | **Control** | 4 | 48 | Yes | SOF/LDV | Yes | 12 |
| **5** | **Control** | 4 | 48 | No | SOF/LDV | No | 8 |
| **6** | **Control** | 4 | 52 | Yes | SOF/SMV | Yes | 12 |
| **7** | **RGT** | 1a | 53 | No | SOF/LDV | No | 8 |
| **8** | **RGT** | 1b | 41 | No | PrOD | No | 12 |
| **9** | **RGT** | 4 | 48 | No | SOF/LDV | No | 12 |

DAA: direct-acting antiviral; RBV: ribavirin; SOF: Sofosbubir; LDV: ledipasvir; DCV: daclastavir; PrOD: paritaprevir-ritonavir/ombitasvir plus dasabuvir; SMV: simeprevir.

**Supplementary table 2**. Patients who achieved SVR12 (percentage) in the RGT and control populations according to the treatment regimen, presence and absence of cirrhosis and HCV genotype.

| | | GT1a | | | GT1b | | | GT3 | | | GT4 | | |
|---|---|---|---|---|---|---|---|---|---|---|---|---|---|
| **Regimen** | **Cirrhosis** | **RGT group** | **Control group** | **p** | **RGT group** | **Control group†** | **p** | **RGT group** | **Control group** | **p** | **RGT group** | **Control group** | **p** |
| **SOF/LDV** | yes | 7 (100) | 31 (96.9) | 1.000 | 3 (100) | 15 (100) | NA | - | 1 (100) | NA | 3 (100) | 16 (94.1) | 1.000 |
| | No | 33 (97.1) | 104 (100) | 0.246 | 16 (100) | 33 (100) | NA | - | 3 (100) | NA | 8 (88.9) | 41 (97.6) | 0.325 |
| **SOF/DCV** | Yes | | | | | | | 1 (100) | 19 (95) | 1.000 | | | |
| | No | | | | | | | 7 (100) | 51 (100) | NA | | | |
| **SOF/VEL** | Yes | | | | | | | 6 (100) | | NA | | | |
| | No | | | | | | | 3 (100) | | NA | | | |
| **SOF/SMV** | Yes | | 5 (100) | NA | | 25 (100) | NA | | | | | 1 (50) | NA |
| | No | | 10 (100) | NA | | 2 (100) | NA | | | | 1 (100) | 4 (100) | |

| | | | | | | | | | | |
|---|---|---|---|---|---|---|---|---|---|---|
| **EBR/GZR** | Yes | 1 (100) | | NA | 1 (100) | 1 (100) | NA | | | |
| | No | 5 (100) | 3 (100) | NA | 1 (100) | 8 (100) | NA | 1 (100) | 4 (100) | NA |
| **PrO** | Yes | | | | | | | 1 (100) | | NA |
| | No | | | | | | | 8 (100) | 15 (100) | NA |
| **PrOD** | Yes | | | | 2 (100) | 18 (100) | NA | | 3 (100) | NA |
| | No | 3 (100) | 17 (100) | NA | 6 (85.7) | 40 (100) | 0.149 | | 7 (100) | NA |

GT: genotype; SVR 12: SVR12: undetectable plasma HCV RNA 12 weeks after the end of therapy; RGT: RAS-guided treatment; SOF: Sofosbubir; RBV: ribavirin; DCV: daclastavir; SMV SMVeprevir; PrOD: paritaprevir-ritonavir/ombitasvir plus dasabuvir; PrO: paritaprevir-ritonavir/ombitasvir; LDV: ledipasvir; VEL: Velpatasvir; EBR: Elbasvir; GZR: grazoprevir; NA: not applicable

†Among 172 individuals with information about cirrhosis status.

**Supplementary table 3**. Comparisons of main characteristics between both the resistance-guided treatment and control groups classified in accordance with the HCV genotype.

| | GT1a | | | GT1b | | | GT3 | | | GT4 | | |
|---|---|---|---|---|---|---|---|---|---|---|---|---|
| **Variables** | **RGT* population n=50** | **Control group n=171** | **p** | **RGT population n=30** | **Control group n=143** | **p** | **RGT population n=17** | **Control group n=75** | **p** | **RGT population n=23** | **Control group n=94** | **p** |
| **SVR12, n (%)** | 49 (98.0) | 170 (99.4) | 0.402 | 29 (96.6) | 143 (100.0) | 0.173 | 17 (100.0) | 74 (98.6) | 1.000 | 22 (95.8) | 91(96.8) | 1.000 |
| **Age*, years** | 49 (46-53) | 50 (46-53) | 0.517 | 55 (49-70) | 52 (41-62) | 0.016 | 49 (46-53) | 52 (46-54) | 0.679 | 48 (46-52) | 49 (47-53) | 0.188 |
| **Male gender, n (%)** | 47 (94) | 129 (75.4) | 0.004 | 20 (66.7) | 71 (49.7) | 0.090 | 14 (82.4) | 60 (80.0) | 0.825 | 20 (87.0) | 75 (79,8) | 0.430 |
| **HIV coinfection, n(%)** | 29 (58.0) | 106 (62) | 0.611 | 12 (43.3) | 30 (21.0) | 0.010 | 13 (76.5) | 39 (52.0) | 0.066 | 20 (87.0) | 64 (68.1) | 0.71 |
| **Cirrhotics, n (%)** | 10 (20) | 47 (27.5) | 0.287 | 10 (33.3) | 38 (26.8) | 0.466 | 10 (58.8) | 23 (30.7) | 0.029 | 6 (26.1) | 24 (25.5) | 0.956 |
| **Pre-treated, n (%)** | 14 (28.0) | 45 (26.3) | 0.813 | 0 (0.0) | 45 (31.5) | <0.001 | 4 (23.5) | 11 (14.7) | 0.372 | 10 (43.5) | 27 (28.7) | 0.173 |
| **RBV use, n (%)** | 9 (18.0) | 30 (17.5) | 0.941 | 3 (10) | 11 (7.7) | 0.673 | 7 (41.2) | 18 (24.0) | 0.151 | 11 (47.8) | 35 (37.2) | 0.351 |
| **8 weeks regimen, n (%)** | 16 (32.0) | 23 (13.5) | 0.002 | 13 (43.3) | 13 (9.1) | <0.001 | 0 (0.0) | 1 (1.3) | 1.000 | 0 (0.0) | 5 (5.3) | 0.258 |

GT: genotype; RGT: Resistance-guided treatment; SVR12: undetectable plasma HCV RNA 12 weeks after the end of therapy; RBV: Ribavirin

*Mean (quartil 1 – quartil 3).
